# Exosomal miR-222 from adriamycin-resistant MCF-7 breast cancer cells promote macrophages M2 polarization via PTEN/Akt to induce tumor progression

**DOI:** 10.18632/aging.202802

**Published:** 2021-03-22

**Authors:** Wei-Xian Chen, Dan-Dan Wang, Bei Zhu, Yi-Zhi Zhu, Lin Zheng, Zhen-Qing Feng, Xi-Hu Qin

**Affiliations:** 1 Department of Breast Surgery, The Affiliated Changzhou No.2 People’s Hospital of Nanjing Medical University, Changzhou 213000, Jiangsu Province, China; 2 Post-Doctoral Working Station, The Affiliated Changzhou No.2 People’s Hospital of Nanjing Medical University, Changzhou 213000, Jiangsu Province, China; 3 Department of Breast Surgery, The First Affiliated Hospital of Nanjing Medical University, Nanjing 210029, Jiangsu Province, China; 4 Department of Pathology, The Affiliated Changzhou No.2 People’s Hospital of Nanjing Medical University, Changzhou 213000, Jiangsu Province, China; 5 Key Laboratory of Antibody Technology, The National Health and Family Planning Commission, Nanjing Medical University, Nanjing 210029, Jiangsu Province, China; 6 Department of General Surgery, The Affiliated Changzhou No.2 People’s Hospital of Nanjing Medical University, Changzhou 213000, Jiangsu Province, China

**Keywords:** breast cancer, exosomes, miR-222, macrophages, microenvironment

## Abstract

Exosome-mediated intercellular communication is considered to be an effective mode for malignant cells to transform biological behaviors in stromal cells. However, the mechanisms by which exosomes modulate macrophages within tumor microenvironment remain largely unclear. In this study, we found that both adriamycin-resistant breast cancer (BCa) cells and the corresponding exosomes (A/exo) were capable of inducing macrophages M2 polarization, which promoted the mobility, proliferation, migration and invasion of BCa cells. Since exosomes deliver microRNAs to affect cellular functions in recipient cells, we confirmed that miR-222 was significantly enriched in A/exo and could be successfully transferred to macrophages. Increased miR-222 level was also detected in exosomes derived from plasma and tissues of chemoresistant patients. Moreover, exosomal miR-222 from A/exo polarized M2 macrophages by targeting PTEN and activating Akt signaling pathway, which promoted BCa cells progression in a feed back loop. Co-culture of adriamycin-resistant BCa cells with macrophages in which miR-222 was upregulated or treated with A/exo facilitated tumor growth *in vivo*. Collectively, our data demonstrated that chemoresistant BCa cells could remodel macrophages within tumor microenvironment by secreting exosomal miR-222, which directly targeted PTEN and caused Akt cascade activation and macrophages M2 polarization. Our findings may provide a foundation for a promising strategy of BCa treatment by targeting exosomes or exosomal miR-222.

## INTRODUCTION

Breast cancer (BCa) is now the most common malignancy among women worldwide [1]. Current therapy options for BCa include surgical resection, systemic chemotherapy, precision radiotherapy, endocrine therapy, and biological targeting agents. However, cancer progression and recurrence are common occurrences following adriamycin-based chemotherapy, which accelerate and usually lead to the development of lung metastasis, liver metastasis, and other distant metastases in many patients [2, 3]. Therefore, a better understanding of molecular mechanisms underlying the progression and metastasis of BCa is crucial for improving patients’ outcomes.

BCa microenvironment is highly complex tissue composed of numerous tumor cells and surrounding stromal cells [4]. Macrophages, the most abundant immune-related stromal cells within tumor microenvironment, display diverse phenotypes and functions [5, 6]. They can be polarized into classic M1 and alternative M2 macrophages in response to various stimuli and cytokines. M1 macrophages characterized by the expressions of pro-inflammatory factors such as IL-12 are potent executive cells that eliminate cancer cells, whereas M2 macrophages produce high levels of anti-inflammatory factors like IL-10 to facilitate tumor growth [5]. Accumulating studies have shown that M2 macrophages could promote chemoresistance, proliferation, invasion and metastasis of tumor cells, and inhibit anti-tumor immune response [7–10]. Due to their contributory effects on cancer progression and metastasis, M2 macrophages have also been proved to be associated with a poor prognosis in BCa patients [11, 12]. Moreover, M2 macrophages are increasingly recognized as prospective therapeutic targets, and attempts are being made to either target molecules that regulate their recruitment or reprogram them toward the M1 phenotype [13, 14]. Although the evidence in support of ongoing dynamic interactions between tumor-macrophages being instrumental in macrophages polarization is gradually mounting [15], the detailed mechanisms and content of communication between macrophages and BCa cells within microenvironment remain largely unclear and warrant an in-depth investigation.

An important mode of intercellular communication may be the bilayer membrane vesicles called exosomes, whose size ranges from 50 to 100 nm in diameter and secreted from diverse cell types [16, 17]. Exosomes act as a vehicle for genetic cargo and constantly deliver a variety of biologically active molecules between heterogeneous populations of cells [18]. Exosome-mediated microRNAs (miRNAs) transfer is particularly interesting, since miRNAs packaged in exosomes could be shuttled into recipient cells and serve as physiologically functional molecules to exert gene silencing activity through a similar mechanism as endogenous miRNAs [19, 20]. Mounting evidence indicates that exosomes from malignant cells deliver miRNAs to remodel tumor microenvironment, thus providing a favourable niche for cancer progression [21]. An example of such includes the exosome-mediated transfer of miR-21 and miR-155 from neuroblastoma to monocytes that resulted in enhanced resistance to chemotherapy [22]. Another study revealed that exosomal miR-23a from hypoxic lung cancer cells increased angiogenesis and vascular permeability by targeting specific genes in endothelial cells [23]. In addition, tumor-secreted miR-9 could be transferred via exosomes to recipient normal fibroblasts, thus inducing the switch to cancer-associated fibroblasts phenotype [24].

Recently, miR-222 has emerged as an important regulator in proliferation, apoptosis, invasion, migration, and chemoresistance of cancer cells through targeting PTEN [25, 26]. As a negative regulator of Akt signaling pathway, PTEN has also been implicated in multiple cellular responses including macrophages polarization [27, 28]. In our previous work, miR-222 was reportedly transferred from adriamycin-resistant BCa cells to sensitive cells by exosomes, where it suppressed cancer cells apoptosis and conferred chemoresistance through targeting PTEN [29]. In the present study, we investigated the role of exosomes in the reprogramming of macrophages. We concluded that exosomes derived from adriamycin-resistant BCa cells could deliver miR-222 to macrophages and induce macrophages M2 polarization, which promoted BCa progression in a feed back loop.

## MATERIALS AND METHODS

### Clinical samples

Plasma samples and tumor tissues were collected from BCa patients who were sensitive (n = 20) or resistant (n = 12) to standard adriamycin-based chemotherapy after surgery between 2017 and 2019 at the Affiliated Changzhou No.2 People’s Hospital of Nanjing Medical University. Chemoresistance was defined by relapse or progression within 12 months or 12 months after the last adriamycin-based chemotherapy. Collection and use of samples was conducted in accordance with ethical principles of the Declaration of Helsinki and approved by the Ethics Committee of Changzhou No.2 People’s Hospital [30]. Informed consent was written by all patients or their guardians prior to this study.

### Cell culture

The MCF-7 BCa cell line was obtained from the Cell Bank of the Chinese Academy of Sciences (Shanghai, China), and the adriamycin-resistant variant (MCF-7/Adr) was established from the parental sensitive cell line (MCF-7/S) by continuous culture in medium containing stepwise increasing concentration of adriamycin in our laboratory as recently reported [31]. They have been validated as appropriate models for studying chemotherapy failure both *in vitro* and *in vivo*. The monocyte cell line THP-1 was purchased from the American Type Culture Collection (ATCC, U.S.A.). THP-1 cells were treated with 100 ng/ml PMA (Sigma-Aldrich, U.S.A.) for differentiation into macrophages for 24 h. After treatment with 100 ng/ml lipopolysaccharide (Sigma, U.S.A.) for 24 h, the cells were polarized into an M1 phenotype. Alternatively, the cells were polarized into an M2 phenotype after treatment with 20 ng/mL interleukin-4 (IL-4, R&D, U.S.A.) for 72 h. In selected experiment, co-culture of macrophages and MCF-7/Adr was performed in 24-well Boyden chambers. Macrophages were seeded on the 0.4-μm inserts, which were permeable to supernatants but not to cellular components. MCF-7/Adr cells were seeded in the lower chambers and grown for 48 h.

All cell lines were cultured in RPMI-1640 medium (HyClone, U.S.A.) supplemented with 10% fetal bovine serum (FBS), 100 U/ml penicillin, and 100 μg/ml streptomycin in a humidified atmosphere of 5% CO_2_ at 37° C. Exosome-free FBS and exosome-depleted medium were prepared by ultracentrifugation (Avanti J-30I, Beckman Coulter, U.S.A.) at 100,000 *g* for 10 h and used for all studies. Testing for mycoplasma contamination was routinely performed by using the Mycoplasma Detection Kit-Quick Test (Biotool, U.S.A.).

### Exosome isolation and identification

A series of centrifugation and ultracentrifugation steps were used to extract exosomes from MCF-7/Adr and MCF-7/S cells. These exosomes were respectively designated as A/exo and S/exo for simplicity. Briefly, cell culture medium was harvested and sequentially centrifuged at 300 *g* for 15 min, at 2,000 *g* for 15 min, and at 12,000 *g* for 30 min to remove floating cells and debris. Following filtration of the resulting supernatants through a 0.22-μm filter, the filtrates were ultracentrifuged at 100,000 *g* (Avanti J-30I, Beckman Coulter, U.S.A.) for 2 h at 4° C to collect exosomal pellets. After resuspended in phosphate-buffered saline (PBS), the isolated exosomes were repeatedly ultracentrifuged at 100,000 *g* for another 2 h at 4° C. Then, exosomes were used immediately or resuspended in PBS and stored at -80° C for further use. Exosomes used in each sample were quantified into 30 μg/ml. In selected experiment, plasma samples were centrifuged at 2,000 *g* for 30 min to remove floating cells and debris. Total Exosome Isolation Reagent (from plasma) (Thermo Fisher, U.S.A.) was added to plasma samples and incubated at 4° C for 30 min. Plasma exosomes were obtained by centrifugation at 10,000 *g* for 10 min and resuspended in PBS.

The shape and size of exosomes were observed using transmission electron microscopy. In short, exosome samples were dissolved in PBS, added dropwisely on a parafilm, and then covered with a copper mesh. After 45 min, the copper mesh was washed by PBS, fixed with 3% glutaraldehyde for 10 min, and finally stained with 2% uranyl acetate solution. Images of exosome morphology were taken with a JEM-1010 electron microscope (JEOL, Japan) at 80 kV.

### Exosome labeling and tracking

Exosome labeling was carried out by using the PKH26 Red Fluorescent Cell Linker Kit (Sigma-Aldrich, U.S.A.) as previously described [29]. Briefly, exosomes were stained with PKH26 red dye at room temperature in the dark for 5 min and blocked with FBS in accordance with the manufacturer’s recommendations. The mixture was subsequently ultracentrifuged at 100,000 *g* for 2 h at 4° C to remove unincorporated dye, washed by PBS, and subjected to additional ultracentrifugation. Then, the final tagged exosomes were resuspended in PBS for further use. Exosomes without PKH26 staining were prepared as negative controls.

Macrophages cultured in a four-chamber slide were treated with PKH26-labeled exosomes or negative controls for 2 h, 12h, and 24 h. After incubation, cells were washed by PBS and fixed in 4% paraformaldehyde solution at room temperature for 10 min. Nucleus staining was performed using the ProLong Gold Antifade Reagent with DAPI (Life Technologies, U.S.A.). Uptake of exosomes by macrophages was then visualized by a confocal laser scanning microscope LSM710 (Carl Zeiss, Germany).

### Luciferase assay and cell transfection

Cells were seeded in 24-well plates with 70% confluence 24 h before transfection. Then, they were co-transfected with 500 ng pMIR-REPORT plasmid (Shanghai GenePharma, China) containing the wild-type PTEN 3’untranslated region (3’UTR) or mutant PTEN 3’UTR, 100 nmol/L miR-222 mimics/inhibitors (Shanghai GenePharma, China) or miRNA negative controls using Lipofectamine 3000 (Invitrogen, U.S.A.). After 48 h transfection, firefly and renilla luciferase activities were measured using the Dual-Luciferase Reporter Assay System (Promega, U.S.A.) according to the manufacturer’s protocols. Renilla luciferase activity was used for normalization.

### Real-time PCR

Exosomal and cellular RNA were extracted using the Total Exosome RNA and Protein Isolation Kit (Invitrogen, U.S.A.) and the mirVana RNA Isolation Kit (Ambion, U.S.A.) according to the manufacturer’s protocols, respectively. Real-time PCR was conducted using the SYBR green technique. Briefly, complementary DNA was synthesized using the SYBR PrimeScript RT-PCR kit (Takara Bio Inc., Japan) on an iCycler iQ system (Bio-Rad, U.S.A.), and templates were subjected to PCR amplification on a LightCycler® 480 System II (Roche, Australia) with the same kit in accordance with the manufacturer’s protocols. The 2^−ΔΔCT^ method was applied to calculate gene expression, and U6 was used for normalization. Primer sequences in the present study were as follows:

CD68 primers, forward: 5’-CCTCCAAGCCCAGATTCAGA-3’,

reverse: 5’-CGCCATGTAGCTCAGGTAGA-3’;

CD80 primers, forward: 5’-CTCTATTGCTGCTCTCTCACT-3’,

reverse: 5’-ATCCCTTTCCTCCTTGACTAC-3’;

CD86 primers, forward: 5’-ACGCGGCTTTTATCTTCACC-3’,

reverse: 5’-GAGGCCGCTTCTTCTTCTTC-3’;

CD163 primers, forward: 5’-ATGCCCATGTTCTTTGCCAG-3’,

reverse: 5’-CAATCTCCCATGTGCTGCTC-3’;

CD206 primers, forward: 5’-TGGACTGGGCTGAATGATGT-3’,

reverse: 5’-TAGCCTCGTTTACTGTCGCA-3’;

and PTEN primers, forward: 5’-TGGCGGAACTTGCAATCCTCAGT-3’,

reverse: 5’-TCCCGTCGTGTGGGTCCTGA-3’.

### Western blotting

Cellular proteins were collected using the RIPA lysis buffer (Biouniquer Technology, China), and protein concentration was analyzed on a Nanodrop 2000 spectrophotometry (Thermo Scientific, U.S.A.). Then, total proteins were separated by SDS-PAGE gel and transferred to polyvinylidene difluoride membranes (Sigma, U.S.A.). After blocking in 5% skim milk for 2 h, membranes were probed with various primary antibodies including CD163, CD206 (1:500, Santa Cruz, U.S.A.), PTEN, total Akt (tAkt), and phosphorylation Akt (pAkt) (1:1000, Cell Signaling, U.S.A.) overnight at 4° C, followed by incubation with horseradish peroxidase-linked secondary antibodies (Cell Signaling, U.S.A.) for 1 h at room temperature. GAPDH (Sigma, U.S.A.) was used for normalization. Bound proteins were visualized using the ECL Plus Kit (Millipore, U.S.A.) with Image Lab Software (Bio-Rad, U.S.A.).

### ELISA assay

Cell culture medium of macrophages treated with or without MCF-7/Adr cells was collected and measured using IL-10 and IL-12 ELISA kits (Ray Biotech, U.S.A.).

### Cell proliferation, mobility, migration, and invasion assay

Cell proliferation was analyzed using the MTS viability assay. Briefly, MCF-7/Adr cells were seeded at a density of 2,000 cells/well in 96-well plates and incubated with 100 μl conditioned medium (50 μl ordinary medium mixed with 50 μl culture medium of exosome-treated macrophages) for 48 h. Afterwards 20 μl MTS solution (Promega, U.S.A.) was added into each well and incubated for 1 h. The absorbance value (optical density) of each well was measured at 490 nm by CliniBio 128 (ASYS-Hitech, Austria).

Cell mobility was assessed using the wound-healing assay. In short, MCF-7/Adr cells were seeded in 24-well plates and cultured with conditioned medium as described above. After 48 h incubation, cells were scratched with the tips of sterile pipettes. Then, the detached cells were washed away by PBS and wounded monolayers were maintained in ordinary medium. Following 12 h incubation, the monolayers were fixed, and images were captured using a fluorescence microscope (Carl Zeiss, Germany), immediately after wounding (0 h) and after culturing (24 h). Relative scratch area was calculated using following equation: (24 h empty area/ 0 h empty area)*100%.

Cell migration and invasion was performed using the transwell assay. For migration assay, MCF-7/Adr cells were seeded on the top chamber. The conditioned medium from differently exosome-treated macrophages were added to the bottom chamber. For invasion assay, the undersides of 8-μm inserts were coated with matrigel (BD Biosciences, U.S.A.) at 37° C overnight before seeding cells. After 24 h incubation, cells that had migrated or invaded to the undersides of the inserts were fixed with 4% paraformaldehyde for 30 min, stained with 1% crystal violet solution, and counted under a microscope in three randomly chosen fields.

### Animal assay

A xenograft assay was carried out to identify the effective role of exosomes in macrophages-mediated breast cancer growth *in vivo*. Female severe combined immunodeficient mice aged 6 weeks were purchased from the Laboratory Animal Center of Nanjing University (Nanjing, China) and maintained under a specific pathogen-free condition. All animal experiments were approved by the Nanjing University Medical Experimental Animal Care Commission and performed according to the institution’s guidelines and animal research principles. Three groups of cell mixture, namely, MCF-7/Adr mixed with PBS-treated macrophages, MCF-7/Adr mixed with S/exo-treated macrophages, and MCF-7/Adr mixed with A/exo-treated macrophages were prepared (5 × 10^5^ tumor cells plus 3 × 10^5^ macrophages per injection) and implanted subcutaneously into the left or right buttocks of the mice. In selected experiment, to evaluate the functional role of exosomal miR-222 in macrophages-mediated breast cancer growth *in vivo*, MCF-7/Adr mixed with macrophages transfected with PBS, miR-NC, or miR-222 mimics were prepared (5 × 10^5^ tumor cells plus 3 × 10^5^ macrophages per injection) and implanted subcutaneously into mice. Tumor volume and mice weight were measured weekly, and tumor growth was observed using a bioluminescence imaging system. Tumor growth curve was plotted using the tumor volume normalized to 7 d on the vertical axis and seeding day on the horizontal axis. After 4 weeks, all mice were sacrificed under general anesthesia, and the transplanted tumors and tissue samples were obtained for further analysis.

### Hematoxylin-eosin (HE) staining

Tissue samples from mice were embedded in paraffin, cut into 8-μm-thick sections, and air-dried for 1 h at 40° C. Then they were stained with HE with the following steps: washed with xylene, treated with 95% ethanol, hydrated in distilled water, stained with hematoxylin, washed by running water, and stained with eosin. After washing and dehydrated with ethanol and xylene, slides were observed under a microscope (Carl Zeiss, Germany).

### Immunohistochemistry (IHC)

Breast cancer tissue samples were fixed, paraffin-embedded, and cut into 8-μm-thick sections. After deparaffinization, rehydration, and antigen retrieval, sections were stained with CD163 antibody overnight at 4° C, followed by incubation with secondary antibody for 30 min at 37° C, and then counterstained with hematoxylin. Stained cells were finally viewed under a fluorescence microscope (Carl Zeiss, Germany).

### Bioinformatics and statistical analysis

Target gene of the selected miRNA was predicted by PicTar (https://pictar.mdc-berlin.de/) which is an online tool for investigating miRNA-mRNA interaction maps [32]. All experiments were carried out at least three times. Data were analyzed using the SPSS 20.0 package, and graphs were generated by the GraphPad software. Differences between groups were compared by Student’s *t* test or by one-way analysis of variance (ANOVA). Pearson *r* correlation was taken for correlation analysis of mRNA expression from TCGA. All the data were expressed as mean ± standard deviation (SD) or median ± range, and *P*<0.05 was considered statistically significant.

## RESULTS

### A/exo induce macrophages M2 polarization

**Figure 1:**
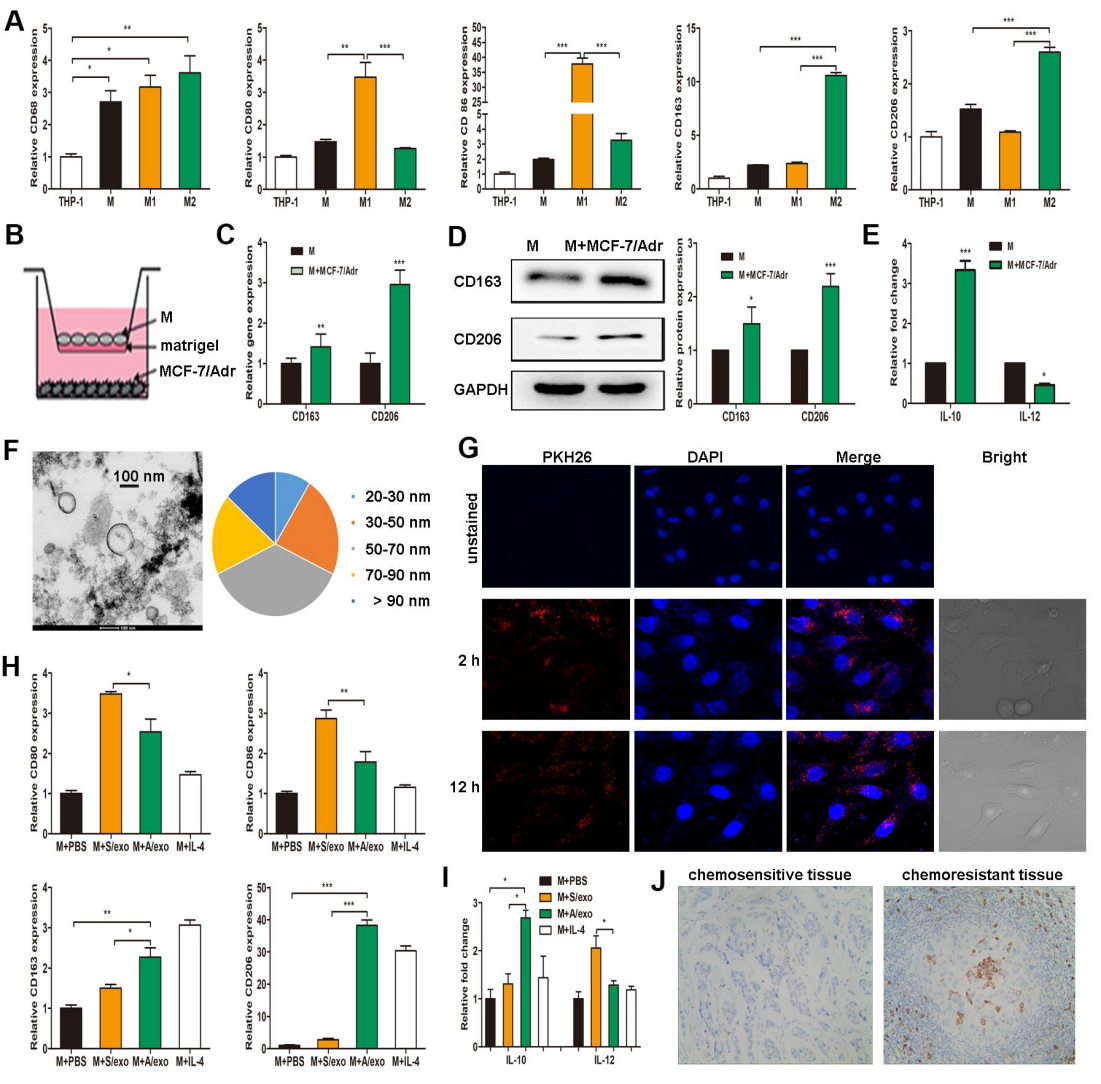
**A/exo derived from MCF-7/Adr cells induce macrophages M2 polarization.** (**A**) Expressions of CD68 (macrophage marker), CD80 and CD86 (M1 phenotype markers), CD163 and CD206 (M2 phenotype markers) were analyzed in THP-1 monocytes and the derived macrophages. (**B**) Macrophages (M) were incubated with MCF-7/Adr cells in a co-culture chamber to avoid direct cell contact. (**C**) After 48 h, M2 phenotype markers CD163 and CD206 were analyzed by PCR. (**D**) CD163 and CD206 were analyzed by western blot. (**E**) IL-10 and IL-12 expression levels in macrophages incubated with MCF-7/Adr cells were evaluated by ELISA. (**F**) Representative transmission electron microscopic image of A/exo showing a spheroid shape with size ranging from 30 to 90 nm (bar indicates 100 nm). (**G**) Representative fluorescence microscopic images showing the uptake of unstained A/exo or PKH26-labeled A/exo (red) by macrophages (blue). (**H**) Macrophages were incubated with A/exo, S/exo, or controls (PBS and IL-4) for 48 h, and expressions of CD80, CD86, CD163, and CD206 were analyzed by PCR. Macrophages treated with IL-4 was used as a positive control. (**I**) IL-10 and IL-12 expression levels in macrophages incubated with A/exo, S/exo, or controls (PBS and IL-4) were evaluated by ELISA. (**J**) Expression of CD163 (grey) was examined by IHC in chemoresistant breast cancer tissues and chemosensitive samples (magnification × 200). Data are shown as mean ± SD, n = 3 independent experiments; * *P*<0.05, ** *P*<0.01, and *** *P*<0.001 compared with controls.

THP-1 monocytes were differentiated into macrophages by a 24 h incubation with PMA. After treatment with lipopolysaccharide, macrophages expressing CD68 were converted to M1 macrophages expressing CD80 and CD86 (M1 phenotype markers). Alternatively, they were polarized into M2 macrophages following treatment with IL-4, expressing CD163 and CD206 (M2 phenotype markers) (Figure 1A). Given that macrophages are the most abundant immune-related stromal cells within the chemoresistant BCa tissues [5, 6], we therefore mimicked such tumor microenvironment by seeding MCF-7/Adr cells (lower) with macrophages (upper) in a co-culture Boyden chamber, which prevented direct cell-cell contact (Figure 1B). The levels of M2 phenotype markers were detected in macrophages, and increased expressions of CD163 and CD206 were found in macrophages co-cultured with MCF-7/Adr, with respect to control macrophages (Figure 1C, 1D). Consistent with the changes in phenotype markers, macrophages co-cultured with MCF-7/Adr exhibited a higher level of IL-10 and a lower level of IL-12 (Figure 1E). These data indicated that MCF-7/Adr cells were able to promote macrophages M2 polarization.

Since exosomes play a significant role in cell-cell communication [18], we hypothesized that exosomes released by MCF-7/Adr might mediate macrophages M2 polarization in our experimental system. After a manner of centrifugation and ultracentrifugation steps, the isolated A/exo from MCF-7/Adr cells were observed by transmission electron microscopy, which revealed the presence of exosomes with similar spheroid morphology and with size ranging from 30 to 90 nm (Figure 1F). Next, to determine whether A/exo could be absorbed by macrophages, we labeled A/exo with PKH26 and the nuclear structure of macrophages with DAPI. Examination with a confocal laser scanning microscope confirmed that a large amount of PKH26-labeled A/exo presenting red fluorescence could be taken up by recipient macrophages along with the culture time, while the cells co-cultured with unstained A/exo only displayed DAPI-induced blue fluorescence (Figure 1G). S/exo showed a homogenous pattern of shape and absorption (not shown).

To evaluate whether A/exo have the potential to promote macrophages M2 polarization, we treated macrophages with PBS, and A/exo or S/exo. The expressions of M1 and M2 phenotype markers were detected in macrophages, and increased trends of CD163 and CD206 whereas reduced levels of CD80 and CD86 were found in macrophages co-cultured with A/exo, with respect to control macrophages incubated with PBS or S/exo (Figure 1H). In addition, A/exo treatment conferred a significant elevation of IL-10 and a reduction of IL-12 relative to PBS or S/exo (Figure 1I). CD163 is associated with the activation of macrophages M2 polarization, and cells expressing CD163 in chemoresistant BCa tissues were much more abundant than those in matched sensitive samples (Figure 1J). Taken together, these findings suggested that A/exo could induce macrophages M2 polarization after absorption by recipient macrophages.

### M2 macrophages induced by A/exo promote BCa progression

**Figure 2:**
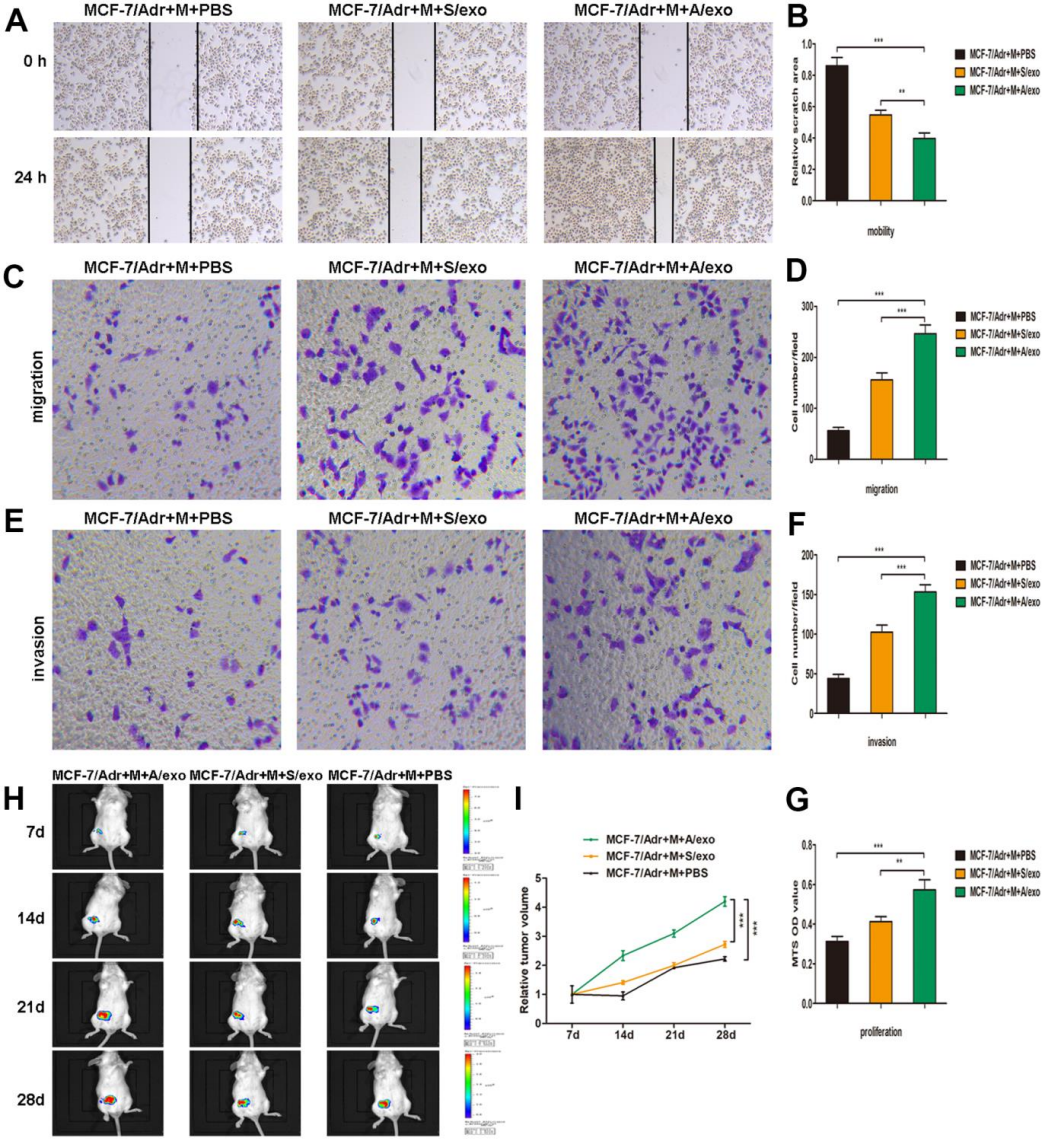
**M2 macrophages induced by A/exo promote the mobility, migration, invasion, and proliferation of MCF-7/Adr cells.** (**A**) MCF-7/Adr cells were incubated with culture medium of macrophages (M) treated with PBS, and S/exo or A/exo. Cell mobility of MCF-7/Adr was observed using the wound-healing assay, and representative images of scratch area were shown. (**B**) Quantitative evaluation of scratch area. (**C**) Cell migration of MCF-7/Adr was evaluated using the transwell assay, and representative images of migrated cells were shown. (**D**) Quantitative evaluation of migrated cells. (**E**) Cell invasion of MCF-7/Adr was determined using the transwell assay, and representative images of invaded cells were shown. (**F**) Quantitative evaluation of invaded cells. (**G**) Cell proliferation of MCF-7/Adr was assessed using the MTS viability assay, and quantitative evaluation of OD values were shown. (**H**) Mice were subcutaneously implanted with a mixture of MCF-7/Adr cells plus macrophages treated with PBS, and S/exo or A/exo. Tumor growth was monitored weekly using a bioluminescence imaging system. (**I**) Tumor volume was recorded weekly, and tumor growth curve was plotted. Data are shown as mean ± SD, n = 3 independent experiments; * *P*<0.05, ** *P*<0.01, and *** *P*<0.001 compared with controls.

To further investigate whether M2 macrophages induced by A/exo could promote BCa progression, we collected the supernatants from macrophages co-cultured with A/exo or S/exo. Wound-healing assay showed the elevated cell mobility of MCF-7/Adr maintained with culture medium from A/exo-treated macrophages in comparison with controls (Figure 2A, 2B). Migration assay revealed that the number of migrating MCF-7/Adr cells remarkably increased when incubated with culture medium from A/exo-stimulated macrophages (Figure 2C, 2D). In addition, invasion assay demonstrated that M2 macrophages induced by A/exo significantly enhanced the invasion ability of MCF-7/Adr cells compared with S/exo (Figure 2E, 2F). MTS assay indicated that treatment with culture medium from A/exo-stimulated macrophages significantly increased viability of MCF-7/Adr cells, with respect to S/exo (Figure 2G). To further evaluate the function of A/exo in macrophages M2 polarization, which could promote BCa progression *in vivo*, MCF-7/Adr cells mixed with A/exo-treated macrophages were implanted subcutaneously into mice. We found that MCF-7/Adr cells mixed with A/exo-treated macrophages generated larger subcutaneous tumors than those generated by MCF-7/Adr mixed with S/exo-treated macrophages (Figure 2H, 2I). These data collectively suggested that activated M2 macrophages induced by A/exo were able to promote BCa cells mobility, migration, invasion, and proliferation.

### A/exo deliver exosomal miR-222 to macrophages

**Figure 3:**
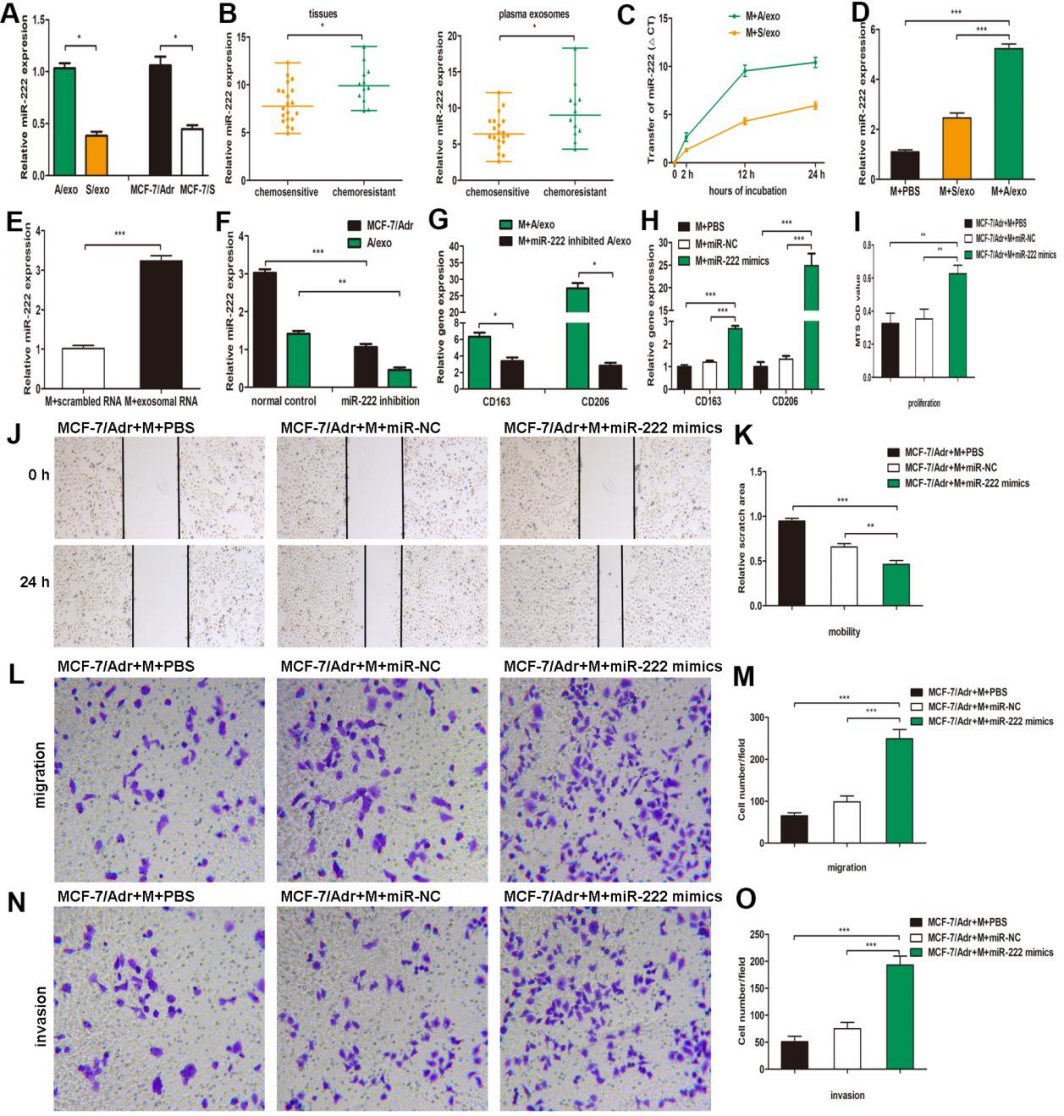
**A/exo deliver exosomal miR-222 to macrophages and promote BCa progression via macrophages M2 polarization.** (**A**) Relative miR-222 expression in A/exo and S/exo was analyzed by PCR. (**B**) Relative miR-222 expression in tissues and plasma-derived exosomes from 20 chemosensitive patients and 12 chemoresistant patients was analyzed by PCR. Data are shown as median ± range. (**C**) Macrophages (M) were incubated with A/exo or S/exo for 0, 2h, 12h, and 24 h and transfer of miR-222 was determined by PCR. Time point 0 stands for macrophages without exosomes. Positive values indicate transfer of miRNA. (**D**) Relative miR-222 expression in macrophages treated with PBS, and S/exo or A/exo for 48 h was analyzed by PCR. (**E**) Relative miR-222 expression in macrophages transfected with total scrambled RNA or total RNA extracted from A/exo for 48 h was evaluated by PCR. (**F**) Relative miR-222 expression in MCF-7/Adr and A/exo after miR-222 inhibition. (**G**) Relative expressions of CD163 and CD206 in macrophages incubated with A/exo, or A/exo from miR-222 inhibited MCF-7/Adr for 48 h were analyzed by PCR. (**H**) Macrophages were transfected with PBS, miR-negative control (miR-NC), or miR-222 mimics for 48 h. Relative expressions of CD163 and CD206 were analyzed by PCR. (**I**) Cell proliferation of MCF-7/Adr was assessed using the MTS viability assay, and quantitative evaluation of OD values were shown. (**J**) Cell mobility of MCF-7/Adr was observed using the wound-healing assay, and representative images of scratch area were shown. (**K**) Quantitative evaluation of scratch area. (**L**) Cell migration of MCF-7/Adr was evaluated using the transwell assay, and representative images of migrated cells were shown. (**M**) Quantitative evaluation of migrated cells. (**N**) Cell invasion of MCF-7/Adr was evaluated using the transwell assay, and representative images of invaded cells were shown. (**O**) Quantitative evaluation of migrated cells. Data are shown as mean ± SD, n = 3 independent experiments; * *P*<0.05, ** *P*<0.01, and *** *P*<0.001 compared with controls.

Accumulating evidence has demonstrated that exosomes serve as a vehicle for genetic cargo and constantly deliver a variety of biologically active molecules including miRNAs between heterogeneous populations of cells, and that exosomal miRNA profiles were miniature maps of their cells of origin [20]. Our recent study has confirmed that miR-222 was highly expressed not only in MCF-7/Adr cells but also in the corresponding A/exo [29]. Here we reinforced our earlier reports that the level of miR-222 in A/exo was markedly increased with respect to S/exo (Figure 3A). We next checked the expressions of miR-222 that were stored in exosomes derived from plasma and BCa tissues. The results showed that exosomal miR-222 was significantly higher in tumor tissues of chemoresistant patients than in those of chemosensitive patients (Figure 3B). Similarly, elevated plasma exosomal miR-222 level was positively associated with chemoresistance status of patients (Figure 3B).

The presence of abundant miR-222 in A/exo prompted us to investigate whether A/exo could deliver miR-222 to target macrophages. Transfer of miR-222 was then determined by PCR, and the variation in Ct values in macrophages incubated with A/exo was evaluated with respect to controls. We found that the abundance of miR-222 elevated progressively in A/exo-treated macrophages along with the culture time (Figure 3C). Macrophages incubated with A/exo expressed higher miR-222 than that with S/exo (Figure 3D). Moreover, miR-222 was present at an increased level in macrophages transfected with total exosomal RNA content from A/exo (Figure 3E). Thus, these suggested that miR-222 was enriched in A/exo and the addition of miR-222 expression could be ascribed to their transport from A/exo to recipient macrophages.

### Exosomal miR-222 promote BCa progression via macrophages M2 polarization

To study the function of exosomal miR-222 in macrophages M2 polarization, we inhibited miR-222 expression in MCF-7/Adr and overexpressed miR-222 in macrophages. The inhibition by miR-222 inhibitors resulted in a reduction of miR-222 in MCF-7/Adr cells and the corresponding A/exo, while the overexpression by miR-222 mimics led to an increase of miR-222 in macrophages, as determined by PCR (Figure 3F). It was also found that M2 phenotype markers CD163 and CD206 were decreased in macrophages cultured with A/exo derived from miR-222-inhibited MCF-7/Adr cells (Figure 3G). Additionally, macrophages transfected with miR-222 mimics displayed increased expressions of CD163 and CD206 (Figure 3H). We then prepared the conditioned medium from macrophages transfected with miR-222 mimics and co-cultured with MCF-7/Adr cells. Consistent with the aforementioned observations, the proliferation, mobility, migration and invasion abilities of MCF-7/Adr cells were significantly enhanced after treatment of conditioned medium from macrophages transfected with miR-222 mimics (Figure 3I–3O). Therefore, exosomal miR-222 could induce macrophages M2 polarization to promote the proliferation, migration and invasion of BCa cells.

### Exosomal miR-222 polarize M2 macrophages via targeting PTEN and activating Akt signaling pathway

**Figure 4:**
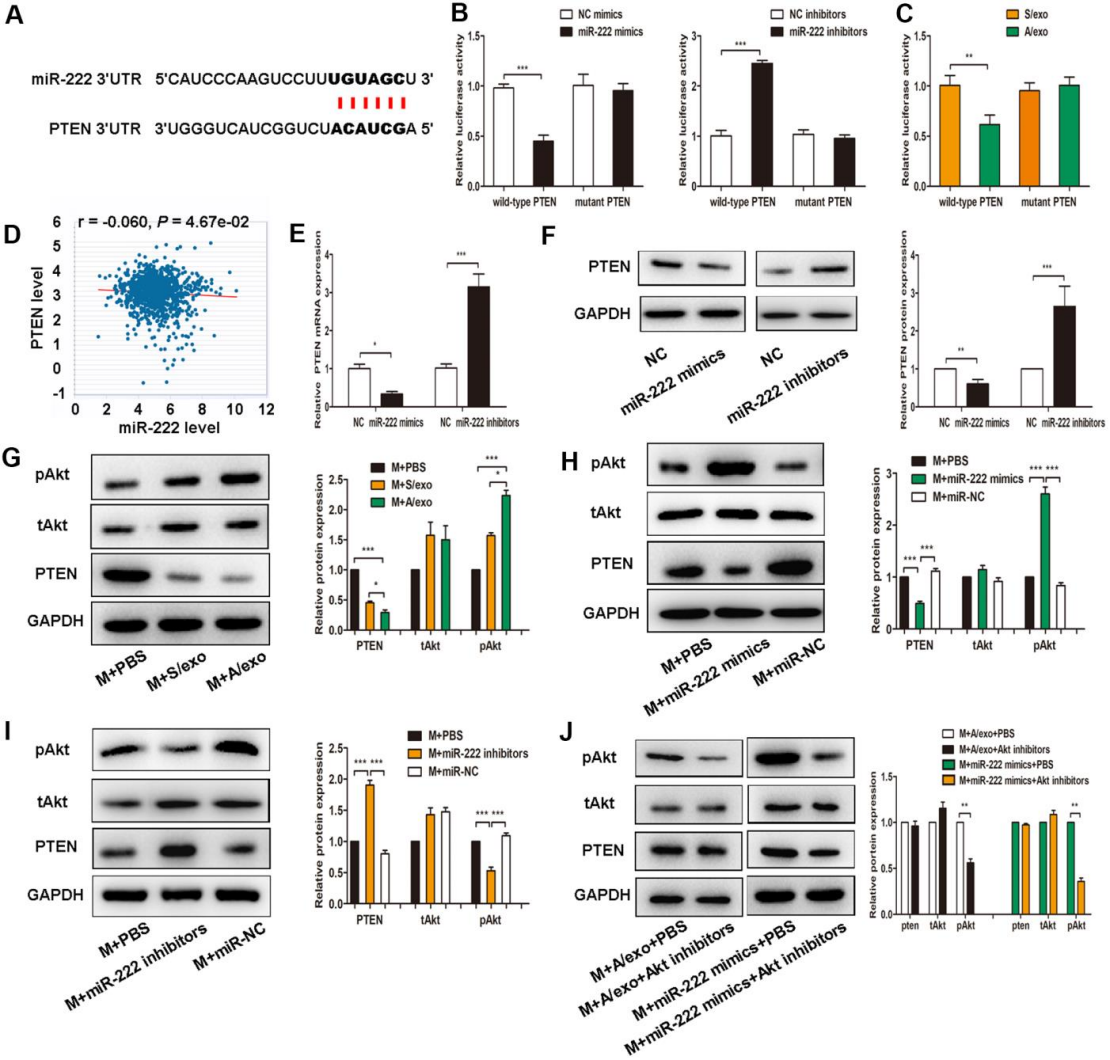
**Exosomal miR-222 induce macrophages M2 polarization via targeting PTEN and activating Akt signaling pathway.** (**A**) The predicted binding sites of miR-222 and PTEN on PicTar online database. (**B**) Luciferase reporter assay in cells transfected with wild-type or mutant PTEN 3’UTR as well as either miR-222 mimics or miR-222 inhibitors were performed. (**C**) Luciferase reporter assay in cells transfected with wild-type or mutant PTEN 3’UTR as well as either A/exo or S/exo were performed. (**D**) PTEN expression is negatively correlated with miR-222 level according to TCGA analysis. (**E**) Relative PTEN mRNA expressions in cells transfected with miR-222 mimics or miR-222 inhibitors were analyzed by PCR. (**F**) Relative PTEN protein expressions in cells transfected with miR-222 mimics or miR-222 inhibitors were analyzed by western blot. (**G**) Expressions of PTEN, tAkt, and pAkt in macrophages (M) treated with PBS, and S/exo or A/exo were analyzed by western blot. (**H**) Expressions of PTEN, tAkt, and pAkt in macrophages transfected with miR-222 mimics or miR-NC were analyzed by western blot. (**I**) Expressions of PTEN, tAkt, and pAkt in macrophages transfected with miR-222 inhibitors or miR-NC were analyzed by western blot. (**J**) Expressions of PTEN, tAkt, and pAkt in macrophages added with A/exo or transfected with miR-222 mimics were analyzed by western blot following Akt inhibitors treatment. Data are shown as mean ± SD, n = 3 independent experiments; * *P*<0.05, ** *P*<0.01, and *** *P*<0.001 compared with controls.

To explore the detailed mechanisms of exosomal miR-222 in macrophages M2 polarization, we predicted its potential target gene PTEN by the online tool PicTar (Figure 4A). Using 3’UTR luciferase reporter assays, we found that miR-222 overexpression significantly inhibited luciferase activity in cells expressing wild-type PTEN 3’UTR reporters, whereas miR-222 inhibitors abolished this suppression (Figure 4B). Moreover, miR-222-containing A/exo effectively reduced luciferase activity in cells expressing wild-type PTEN 3’UTR reporters with respect to mutant PTEN 3’UTR reporters (Figure 4C). PTEN expression is negatively correlated with miR-222 level according to TCGA analysis (Figure 4D). Coincidently, overexpression of miR-222 by mimics significantly decreased PTEN expression in macrophages, while miR-222 inhibition revealed an opposite effect (Figure 4E, 4F).

Recent studies have indicated that activation of Akt signaling pathway participates in macrophages M2 polarization [27, 28], we therefore sought to investigate whether exosomal miR-222 polarized M2 macrophages via activating Akt signaling pathway. It was found that expression of pAkt in macrophages treated with A/exo was higher than that in macrophages treated with S/exo (Figure 4G). We next transfected macrophages with miR-222 mimics or miR-222 inhibitors and checked the activation of Akt signaling pathway. Overexpression of miR-222 in macrophages significantly reduced PTEN level and elevated pAkt expression (Figure 4H), while miR-222 inhibitors showed an opposite effect (Figure 4I). However, Akt inhibitor LY294002 suppressed the M2 polarization induced by miR-222 mimics and A/exo (Figure 4J). These data suggested that exosomal miR-222 activated Akt signaling pathway to promote macrophages M2 polarization via targeting PTEN in macrophages.

### Exosomal miR-222 stimulate tumor growth via macrophages M2 polarization and pre-metastatic niche formation *in vivo*

**Figure 5:**
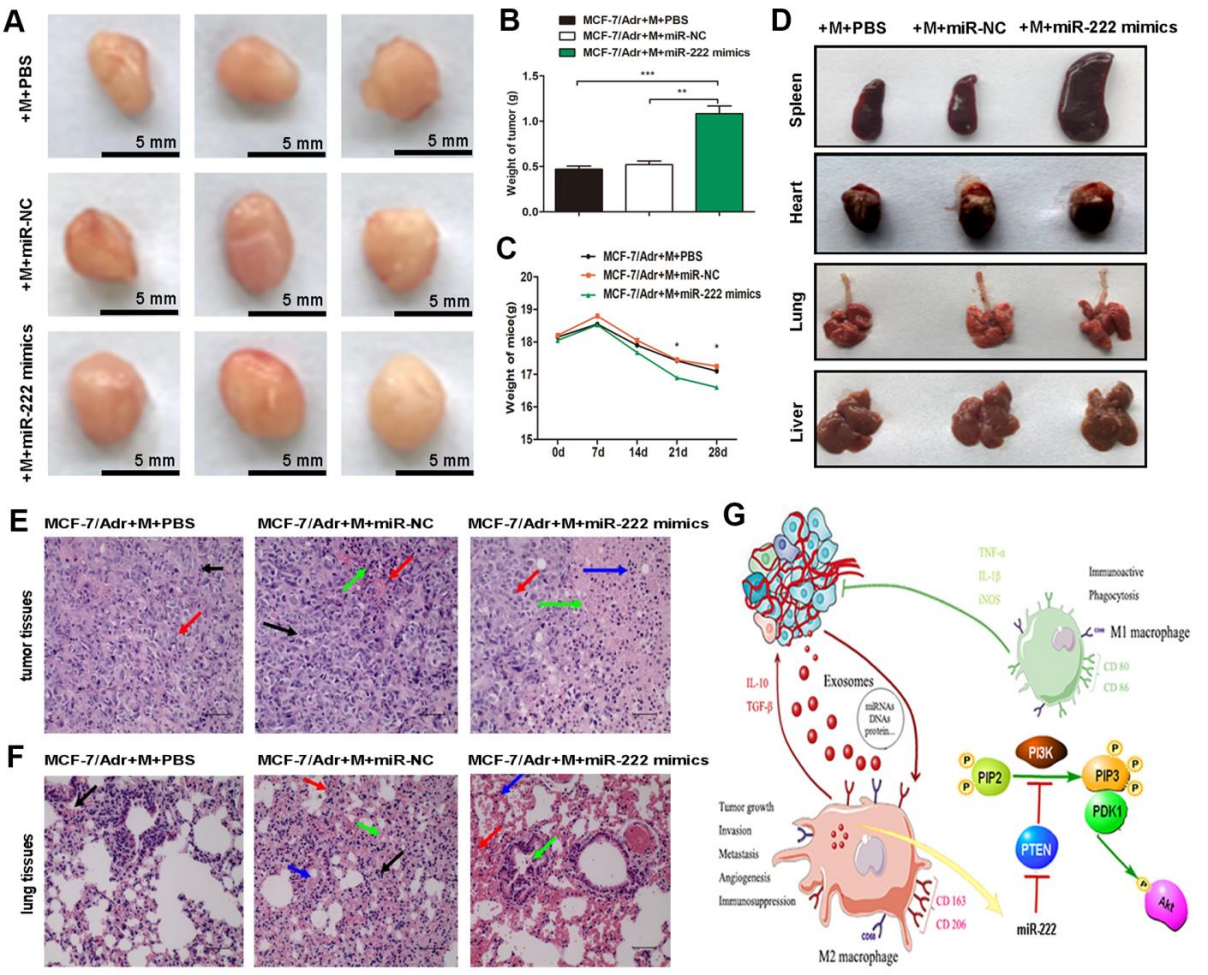
**Exosomal miR-222 stimulate tumor growth via macrophages M2 polarization and pre-metastatic niche formation *in vivo*.** (**A**) MCF-7/Adr mixed with macrophages transfected with miR-222 mimics or miR-NC were implanted subcutaneously into mice, and transplanted tumors and tissue samples were collected 4 weeks later. Representative images of subcutaneous tumors were shown (bar indicates 5 mm). (**B**) Weight of tumor was measured. (**C**) Weight of mice was monitored weekly. (**D**) Appearance of tissue samples including spleen, heart, lung, and liver were shown. (**E**) Representative HE staining images (magnification × 200) of tumor tissues showing the fibrous tissue separation (black arrow), malignant cell proliferation (red arrow), necrosis area (green arrow), and macrophages infiltration (blue arrow). (**F**) Representative HE staining images (magnification × 200) of lung tissues showing the macrophages infiltration (black arrow), defuse hemorrhage (red arrow), stromal cells necrosis (blue arrow), and bronchus exudation (green arrow). (**G**) Schematic model of exosomal miR-222 within A/exo promoting macrophages M2 polarization and breast cancer metastasis. Data are shown as mean ± SD, n = 3 independent experiments; * *P*<0.05, ** *P*<0.01, and *** *P*<0.001 compared with controls.

Next, we determined whether exosomal miR-222 stimulated tumor formation *in vivo*. MCF-7/Adr mixed with macrophages transfected with miR-222 mimics were prepared and implanted subcutaneously into mice, and the transplanted tumors and tissue samples were collected 4 weeks later. Versus those treated with MCF-7/Adr mixed with macrophages transfected with controls, the volume and weight of subcutaneous tumors in MCF-7/Adr mixed with macrophages transfected with miR-222 mimics group were increased (Figure 5A, 5B), whereas the weight of mice in MCF-7/Adr cells mixed with macrophages transfected with miR-222 mimics group was significantly reduced (Figure 5C). Although we failed to obtain any nodules of metastatic tumors in lung and liver, we observed that the spleen, heart, and lung were larger in MCF-7/Adr cells mixed with macrophages transfected with miR-222 mimics group with respect to control groups (Figure 5D). Histomorphology of the obtained tumors and lung tissues were further observed by using HE staining. We found that the malignant cell proliferation (red arrow), necrosis area (green arrow), and macrophages infiltration (blue arrow) were increased in mice treated with MCF-7/Adr mixed with macrophages transfected with miR-222 mimics group in respect to control groups, suggesting the accelerated tumor cell growth (Figure 5E). Moreover, there was an increase of defuse hemorrhage (red arrow), stromal cells necrosis (blue arrow), and bronchus exudation (green arrow) in mice treated with MCF-7/Adr mixed with macrophages transfected with miR-222 mimics group, suggesting a gradual generation of lung pre-metastatic niche (Figure 5F). These results suggested that exosomal miR-222 stimulated tumor growth via macrophages M2 polarization and pre-metastatic niche formation *in vivo*.

## DISCUSSION

There is a growing number of evidence suggesting the BCa treatment remains a challenge, due to the inevitable progression of resistance to first-line chemotherapy with adriamycin and susceptibility to early recurrence and metastasis [2, 3]. Therefore, investigating the molecular mechanisms underlying the progression and metastasis may be of great significance for improving BCa patients’ outcomes. Researches over the past few years have shown that exosomal miRNAs play critical roles in mediating cell-cell communication, especially between cancer cells and stromal cells [18, 20]. In this study, we found that exosomal miR-222 could be transferred from chemoresistant BCa cells to macrophages, resulting in enhancement of macrophages M2 polarization through the suppression of miR-222’s target PTEN.

Exosome-mediated intercellular communication is considered to be an effective mode to transform biological behaviors in recipient cells [18, 19]. In our previous study, we have demonstrated that chemoresistant BCa cells may spread resistance capacity to sensitive cells by transmitting exosomes and that such effects could be attributed to the constant transfer of specific miRNAs [29]. Macrophages have dual influences on cancer development according to the activation status, and the anti-tumor and pro-tumor effects are respectively induced by classical M1 and alternatively activated M2 phenotypes [5]. Considering the evidence that BCa mass is comprised mostly of stromal cells, it is also possible for chemoresistant cancer cells to communicate with macrophages by transferring miRNAs via exosomes. Therefore, we used a transwell system to mimic tumor microenvironment; this system allowed MCF-7/Adr cells to communicate with macrophages without direct contact. In the present work, macrophages co-cultured with MCF-7/Adr exhibited higher levels of M2 phenotype markers. Moreover, we found that A/exo derived from MCF-7/Adr cells could be taken up by macrophages and were capable of inducing macrophages M2 polarization, which promoted the proliferation, mobility, migration and invasion of BCa cells. As a matter of fact, MCF-7 cell line is a luminal subtype of breast cancer. Remarkably, we have already compared the miRNA expression profiles of drug-resistance MDA-MB-231 breast cancer cells (a triple negative subtype of breast cancer) and their exosomes. According to our unpublished data, exosomal miRNA derived from chemo-resistant MDA-MB-231 breast cancer cells could induce macrophages M2 polarization, but the detailed mechanism remains to be investigated. Our observation, which was consistent with previous reports of communication between tumor cells and surrounding stromal cells [22–24, 28], would add another piece of evidence to the emerging idea that exosomes could create an immune suppression niche promoting tumor progression [21].

Among the genetic cargo carried by exosomes are a class of small noncoding RNA known as miRNAs, which regulate gene expression post-transcriptionally by binding to the 3’UTR of target mRNAs to either inhibit translation or induce degradation [33]. The oncogenic role of miR-222 has been well reviewed in BCa, where the effects of miR-222 in facilitating cell proliferation, angiogenesis, invasion and metastasis, and chemoresistance were documented [25]. Chemoresistant BCa tissues and cell lines have also been found to display up-regulated miR-222 expression [26]. Here we demonstrated that miR-222 was significantly enriched in A/exo and could be successfully transferred from MCF-7/Adr cells to macrophages. The overexpression of miR-222 increased macrophages M2 polarization, which was followed by the enhanced proliferation, mobility, migration and invasion abilities of MCF-7/Adr cells. In addition, we showed that miR-222 levels were increased in exosomes derived from plasma and tissues of chemoresistant patients than in those of chemosensitive patients. Albeit speculative, it seems plausible that horizontal transfer of exosomal miR-222 from chemoresistant cells could not only dictate their own microenvironment for the benefit of cancer progression but also spread malignant capacity over a distance. Remarkably, a number of miRNAs have been reported in the control of macrophages activation [34]. Macrophages-targeted miRNA delivery has been preliminary evaluated for anti-cancer therapy, raising the possibility that tumor suppression can be achieved through the repolarization of M2 macrophages to M1 macrophages [13, 35]. Thus, targeting miR-222 might be a promising strategy.

To further probe into the mechanism, specific binding sites between miR-222 and the 3’UTR of PTEN were confirmed using bioinformatics analysis, which was also verified in multiple malignancies [36–38]. PTEN, the most frequently altered tumor suppressor genes in human cancers including BCa, was known to suppress cell survival signaling, such as Akt cascade, thereby accelerating cell death [39]. In our previous experiments, miR-222-mediated PTEN reduction led to increased PI3K/Akt signal activation and reduced sensitivity of BCa cells to agent exposure, suggesting Akt may be involved in miR-222/PTEN pathway [37, 38]. A growing number of studies have recently reported that PTEN is essential in immunity modulation by regulating PI3K/Akt axis and that Akt activation is crucial for macrophages M2 polarization [27, 28, 40]. In the present work, we found that A/exo inhibited PTEN expression in macrophages and then activated Akt pathway. MiRNAs regulate gene expression by binding to the 3’UTR of target mRNAs [33]. Our data also showed that miR-222 could target the 3’UTR of PTEN and inhibit its expression. Then decreased expression of PTEN, inhibited by miR-222, resulted in phosphorylation of Akt and activation of Akt signaling pathway. Therefore, our results suggested that chemoresistant BCa cells decreased PTEN level in macrophages within tumor microenvironment by secreting exosomal miR-222, which directly targeted PTEN and caused Akt cascade activation and macrophages M2 polarization. Particularly in our recent work, miR-222, released by chemoresistant BCa cells, was shown to transport to sensitive cells via exosomes and suppressed PTEN expression, which contributed to enhanced cell survival and drug insensitivity [29]. Our series of studies about exosome-mediated miRNA delivery in intercellular communication would lend support to a putative mechanism whereby chemoresistant BCa cells not only provide the sensitive ones a survival advantage to adapt to toxic insult but also switch macrophages from tumor-suppressing to tumor-promoting by delivering exosomal miR-222.

In summary, we have demonstrated that exosomal miR-222 derived from chemoresistant BCa cells induced macrophages M2 polarization by activation of PTEN/Akt pathway, which promoted BCa cells progression in a feed back loop (Figure 5G). Our findings may provide a foundation for a promising strategy of BCa treatment by targeting exosomes or exosomal miR-222.

### Ethics approval and consent to participate

Collection and use of clinical samples was conducted in accordance with ethical principles of the Declaration of Helsinki and approved by the Ethics Committee of Changzhou No.2 People’s Hospital. Informed consent was written by all patients or their guardians prior to this study. All institutional and national guidelines for the care and use of laboratory animals were followed.

### Availability of data and material

The data sets analyzed during the current study are available from the corresponding author on reasonable request.
